# 1,5-Bis(4-meth­oxy­benzyl­idene)thio­carbonohydrazide methanol monosolvate

**DOI:** 10.1107/S1600536811029035

**Published:** 2011-07-23

**Authors:** Xinyu Zhao

**Affiliations:** a School of Petrochemical Engineering, Shenyang University of Technology, Liaoyang 111003, People’s Republic of China

## Abstract

In the title compound, C_17_H_18_N_4_O_2_S·CH_3_OH, the two benzene rings in the thio­carbonohydrazide mol­ecule form a dihedral angle of 22.42 (18)°. Pairs of N—H⋯S hydrogen bonds link thio­carbonohydrazide mol­ecules into centrosymmetric dimers. Methanol solvent mol­ecules serve as donors (O—H⋯S and O—H⋯N) and acceptors (N—H⋯O and C—H⋯O) of weak inter­molecular hydrogen bonds, which link further these dimers into double ribbons along the *b* axis.

## Related literature

For related Schiff base derivatives of thio­carbohydrazide, see: Loncle *et al.* (2004 ▶); Camp *et al.* (2010 ▶); Opstal & Verpoort (2003 ▶). For a related structure, see: Affan *et al.* (2010 ▶).
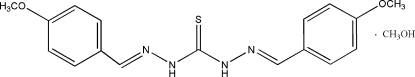


## Experimental

### 

#### Crystal data

C_17_H_18_N_4_O_2_S·CH_4_O
*M* *_r_* = 374.46
Triclinic, 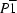

*a* = 8.8021 (6) Å
*b* = 9.9949 (10) Å
*c* = 11.5902 (13) Å
α = 83.132 (1)°
β = 84.179 (2)°
γ = 70.505 (1)°
*V* = 952.24 (16) Å^3^
*Z* = 2
Mo *K*α radiation
μ = 0.20 mm^−1^
*T* = 298 K
0.42 × 0.39 × 0.32 mm

#### Data collection

Bruker SMART APEX CCD area-detector diffractometer
Absorption correction: multi-scan (*SADABS*; Sheldrick, 1996 ▶) *T*_min_ = 0.923, *T*_max_ = 0.940
4936 measured reflections
3302 independent reflections
1934 reflections with *I* > 2σ(*I*)
*R*_int_ = 0.023

#### Refinement

*R*[*F* ^2^ > 2σ(*F* ^2^)] = 0.047
*wR*(*F* ^2^) = 0.137
*S* = 1.01
3302 reflections
239 parameters
H-atom parameters constrained
Δρ_max_ = 0.21 e Å^−3^
Δρ_min_ = −0.18 e Å^−3^

### 

Data collection: *SMART* (Bruker, 2007 ▶); cell refinement: *SAINT* (Bruker, 2007 ▶); data reduction: *SAINT*; program(s) used to solve structure: *SHELXS97* (Sheldrick, 2008 ▶); program(s) used to refine structure: *SHELXL97* (Sheldrick, 2008 ▶); molecular graphics: *SHELXTL* (Sheldrick, 2008 ▶); software used to prepare material for publication: *SHELXTL*.

## Supplementary Material

Crystal structure: contains datablock(s) I, global. DOI: 10.1107/S1600536811029035/cv5128sup1.cif

Structure factors: contains datablock(s) I. DOI: 10.1107/S1600536811029035/cv5128Isup2.hkl

Supplementary material file. DOI: 10.1107/S1600536811029035/cv5128Isup3.cml

Additional supplementary materials:  crystallographic information; 3D view; checkCIF report

## Figures and Tables

**Table 1:** Hydrogen-bond geometry (Å, °)

*D*—H⋯*A*	*D*—H	H⋯*A*	*D*⋯*A*	*D*—H⋯*A*
O3—H3*A*⋯N1	0.82	2.55	3.171 (3)	134
O3—H3*A*⋯S1	0.82	2.58	3.346 (3)	156
N2—H2⋯O3^i^	0.86	2.45	3.174 (3)	142
N3—H3⋯S1^ii^	0.86	2.61	3.446 (3)	165
C2—H2*A*⋯O3^i^	0.93	2.51	3.300 (4)	143

Symmetry codes: (i) 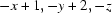; (ii) 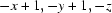.

**Fig. 1.**
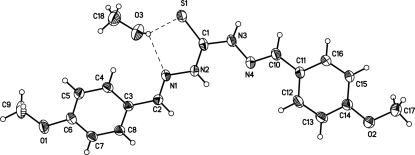
View of (I) showing the atomic numbering and 30% probability displacement ellipsoids. Dashed lines denote hydrogen bonds.

## supplementary crystallographic information

### Comment

In recent years, there has been considerable interest in the chemistry of thiocarbohydrazide Schiff base derivatives (Loncle *et al.*, 2004; Camp *et al.*, 2010),  because these derivatives offer opportunities for tuning the metal centred electronic factor, enhancing the solubility and stability of either homogeneous or heterogeneous catalysts (Opstal *et al.*, 2003). Herein we present the title compound, (I).

In (I) (Fig. 1), the bond lengths and angles are normal and correspond to those observed in 1,5-bis[(*E*)-1-(2-hydroxyphenyl)ethylidene] thiocarbonohydrazide monohydrate (Affan *et al.*, 2010). Four N atoms and the C=S are almost coplanar, the N1/N2/C2 plane and the benzene ring C3–C8 form a dihedral angle of 12.32 (3)°. The benzene rings C3–C8 and C11–C16 form a dihedral angle of 22.42 (18) °.

In the crystal structure, intermolecular N—H···S hydrogen bonds (Table 1) link the molecules into centrosymmetric dimers. Solvent molecules serve as donors [O—H···S and O—H···N] and acceptors [N—H···O and C—H···O] of the weak intermolecular hydrogen bonds (Table 1), which link further these dimers into doubled ribbons along axis *b*.

### Experimental

4-Methoxybenzaldehyde (10.0 mmol), 30 ml e thanol and thiocarbohydrazide (5.0 mmol) were mixed in 50 ml flash After stirring 3 h at 373 K, the resulting mixture was cooled to room temperature, and recrystalized from ethanol, and afforded the title compound as a crystalline solid.

### Refinement

All H atoms were placed in geometrically idealized positions (N—H 0.86; O—H 0.82 and C—H 0.93–0.96 Å) and treated as riding on their parent atoms, with *U*_iso_(H) = 1.2–1.5*U*_eq_ of the parent atom.

### Crystal data

**Table:**

C_17_H_18_N_4_O_2_S·CH_4_O	*Z* = 2
*M**_r_* = 374.46	*F*(000) = 396
Triclinic, *P*1	*D*_x_ = 1.306 Mg m^−^^3^
*a* = 8.8021 (6) Å	Mo *K*α radiation, λ = 0.71073 Å
*b* = 9.9949 (10) Å	Cell parameters from 1379 reflections
*c* = 11.5902 (13) Å	θ = 2.7–25.2°
α = 83.132 (1)°	µ = 0.20 mm^−^^1^
β = 84.179 (2)°	*T* = 298 K
γ = 70.505 (1)°	Block, red
*V* = 952.24 (16) Å^3^	0.42 × 0.39 × 0.32 mm

### Data collection

**Table:**

Bruker SMART APEX CCD area-detector diffractometer	3302 independent reflections
Radiation source: fine-focus sealed tube	1934 reflections with *I* > 2σ(*I*)
graphite	*R*_int_ = 0.023
phi and ω scans	θ_max_ = 25.0°, θ_min_ = 2.7°
Absorption correction: multi-scan (*SADABS*; Sheldrick, 1996)	*h* = −7→10
*T*_min_ = 0.923, *T*_max_ = 0.940	*k* = −11→11
4936 measured reflections	*l* = −13→12

### Refinement

**Table:**

Refinement on *F*^2^	Primary atom site location: structure-invariant direct methods
Least-squares matrix: full	Secondary atom site location: difference Fourier map
*R*[*F*^2^ > 2σ(*F*^2^)] = 0.047	Hydrogen site location: inferred from neighbouring sites
*wR*(*F*^2^) = 0.137	H-atom parameters constrained
*S* = 1.01	*w* = 1/[σ^2^(*F*_o_^2^) + (0.0585*P*)^2^ + 0.1975*P*] where *P* = (*F*_o_^2^ + 2*F*_c_^2^)/3
3302 reflections	(Δ/σ)_max_ < 0.001
239 parameters	Δρ_max_ = 0.21 e Å^−^^3^
0 restraints	Δρ_min_ = −0.18 e Å^−^^3^

### Special details

**Table:**

Geometry. All e.s.d.'s (except the e.s.d. in the dihedral angle between two l.s. planes) are estimated using the full covariance matrix. The cell e.s.d.'s are taken into account individually in the estimation of e.s.d.'s in distances, angles and torsion angles; correlations between e.s.d.'s in cell parameters are only used when they are defined by crystal symmetry. An approximate (isotropic) treatment of cell e.s.d.'s is used for estimating e.s.d.'s involving l.s. planes.
Refinement. Refinement of *F*^2^ against ALL reflections. The weighted *R*-factor *wR* and goodness of fit *S* are based on *F*^2^, conventional *R*-factors *R* are based on *F*, with *F* set to zero for negative *F*^2^. The threshold expression of *F*^2^ > σ(*F*^2^) is used only for calculating *R*-factors(gt) *etc*. and is not relevant to the choice of reflections for refinement. *R*-factors based on *F*^2^ are statistically about twice as large as those based on *F*, and *R*- factors based on ALL data will be even larger.

### Fractional atomic coordinates and isotropic or equivalent isotropic displacement parameters (Å^2^)

**Table:**

	*x*	*y*	*z*	*U*_iso_*/*U*_eq_	
S1	0.63183 (11)	0.63686 (9)	−0.08592 (7)	0.0604 (3)	
N1	0.6991 (3)	0.8790 (2)	−0.00620 (19)	0.0435 (6)	
N2	0.6220 (3)	0.8089 (2)	0.07669 (19)	0.0445 (6)	
H2	0.5951	0.8368	0.1454	0.053*	
N3	0.5206 (3)	0.6273 (2)	0.1308 (2)	0.0487 (7)	
H3	0.5021	0.5523	0.1154	0.058*	
N4	0.4772 (3)	0.6718 (2)	0.2409 (2)	0.0458 (6)	
O1	1.0808 (3)	1.2665 (2)	−0.28491 (19)	0.0653 (7)	
O2	0.1682 (3)	0.6793 (2)	0.76724 (19)	0.0688 (7)	
C1	0.5902 (3)	0.6955 (3)	0.0468 (2)	0.0424 (7)	
C2	0.7256 (3)	0.9876 (3)	0.0215 (2)	0.0412 (7)	
H2A	0.6890	1.0203	0.0944	0.049*	
C3	0.8135 (3)	1.0612 (3)	−0.0612 (2)	0.0388 (7)	
C4	0.8918 (4)	1.0014 (3)	−0.1615 (2)	0.0471 (8)	
H4A	0.8839	0.9150	−0.1773	0.057*	
C5	0.9813 (4)	1.0662 (3)	−0.2389 (3)	0.0522 (8)	
H5	1.0332	1.0239	−0.3057	0.063*	
C6	0.9927 (4)	1.1946 (3)	−0.2158 (3)	0.0463 (8)	
C7	0.9135 (3)	1.2564 (3)	−0.1173 (3)	0.0473 (8)	
H7	0.9189	1.3442	−0.1029	0.057*	
C8	0.8264 (3)	1.1902 (3)	−0.0399 (2)	0.0439 (7)	
H8	0.7757	1.2323	0.0272	0.053*	
C9	1.1754 (5)	1.1998 (4)	−0.3814 (3)	0.0838 (12)	
H9A	1.1057	1.1927	−0.4373	0.126*	
H9B	1.2437	1.1062	−0.3555	0.126*	
H9C	1.2410	1.2554	−0.4168	0.126*	
C10	0.3907 (4)	0.6071 (3)	0.3027 (3)	0.0501 (8)	
H10	0.3638	0.5391	0.2686	0.060*	
C11	0.3309 (4)	0.6312 (3)	0.4222 (2)	0.0458 (8)	
C12	0.3712 (4)	0.7200 (3)	0.4869 (3)	0.0589 (9)	
H12	0.4377	0.7710	0.4525	0.071*	
C13	0.3152 (4)	0.7345 (3)	0.6009 (3)	0.0639 (10)	
H13	0.3445	0.7943	0.6433	0.077*	
C14	0.2149 (4)	0.6601 (3)	0.6531 (3)	0.0498 (8)	
C15	0.1720 (4)	0.5731 (3)	0.5907 (3)	0.0540 (8)	
H15	0.1037	0.5236	0.6248	0.065*	
C16	0.2307 (4)	0.5589 (3)	0.4766 (3)	0.0573 (9)	
H16	0.2017	0.4984	0.4347	0.069*	
C17	0.0688 (5)	0.6007 (4)	0.8248 (3)	0.0738 (11)	
H17A	0.1258	0.5005	0.8237	0.111*	
H17B	−0.0289	0.6249	0.7854	0.111*	
H17C	0.0429	0.6239	0.9040	0.111*	
O3	0.5382 (3)	0.9498 (3)	−0.2485 (2)	0.0849 (8)	
H3A	0.5673	0.8888	−0.1940	0.127*	
C18	0.6365 (5)	0.9076 (5)	−0.3470 (3)	0.0884 (13)	
H18A	0.5743	0.9401	−0.4144	0.133*	
H18B	0.6812	0.8055	−0.3413	0.133*	
H18C	0.7224	0.9477	−0.3540	0.133*	

### Atomic displacement parameters (Å^2^)

**Table:**

	*U*^11^	*U*^22^	*U*^33^	*U*^12^	*U*^13^	*U*^23^
S1	0.0755 (7)	0.0533 (5)	0.0593 (5)	−0.0349 (5)	0.0242 (5)	−0.0148 (4)
N1	0.0458 (15)	0.0437 (14)	0.0427 (14)	−0.0205 (12)	0.0033 (12)	0.0020 (11)
N2	0.0531 (16)	0.0473 (14)	0.0359 (13)	−0.0236 (13)	0.0047 (12)	−0.0001 (11)
N3	0.0550 (16)	0.0413 (14)	0.0514 (15)	−0.0228 (13)	0.0114 (13)	−0.0019 (12)
N4	0.0468 (16)	0.0451 (14)	0.0437 (15)	−0.0162 (13)	0.0036 (12)	0.0016 (12)
O1	0.0743 (16)	0.0593 (14)	0.0680 (15)	−0.0371 (13)	0.0204 (13)	−0.0045 (12)
O2	0.0862 (18)	0.0785 (16)	0.0553 (14)	−0.0468 (14)	0.0218 (13)	−0.0227 (12)
C1	0.0329 (17)	0.0382 (16)	0.0516 (18)	−0.0106 (14)	0.0059 (14)	0.0037 (14)
C2	0.0401 (18)	0.0455 (17)	0.0389 (16)	−0.0152 (14)	−0.0007 (14)	−0.0048 (13)
C3	0.0348 (16)	0.0412 (16)	0.0418 (16)	−0.0138 (13)	−0.0008 (13)	−0.0056 (13)
C4	0.0506 (19)	0.0429 (17)	0.0529 (19)	−0.0220 (15)	0.0043 (16)	−0.0104 (15)
C5	0.059 (2)	0.0518 (19)	0.0490 (18)	−0.0235 (17)	0.0127 (16)	−0.0134 (15)
C6	0.0450 (19)	0.0436 (18)	0.0522 (19)	−0.0202 (15)	−0.0008 (15)	0.0028 (15)
C7	0.0495 (19)	0.0382 (17)	0.0580 (19)	−0.0193 (15)	0.0007 (16)	−0.0082 (15)
C8	0.0434 (18)	0.0430 (17)	0.0474 (18)	−0.0158 (15)	0.0011 (15)	−0.0109 (14)
C9	0.087 (3)	0.075 (3)	0.084 (3)	−0.033 (2)	0.040 (2)	−0.004 (2)
C10	0.056 (2)	0.0481 (18)	0.0496 (19)	−0.0248 (16)	−0.0010 (16)	0.0049 (15)
C11	0.0470 (19)	0.0447 (17)	0.0461 (18)	−0.0194 (15)	0.0039 (15)	0.0011 (14)
C12	0.067 (2)	0.054 (2)	0.065 (2)	−0.0360 (18)	0.0159 (19)	−0.0095 (17)
C13	0.077 (3)	0.060 (2)	0.068 (2)	−0.041 (2)	0.014 (2)	−0.0211 (17)
C14	0.053 (2)	0.0495 (18)	0.0498 (19)	−0.0210 (16)	0.0049 (16)	−0.0088 (15)
C15	0.060 (2)	0.063 (2)	0.0486 (19)	−0.0372 (18)	0.0078 (16)	−0.0024 (16)
C16	0.074 (2)	0.063 (2)	0.049 (2)	−0.0430 (19)	0.0036 (18)	−0.0061 (16)
C17	0.089 (3)	0.092 (3)	0.052 (2)	−0.049 (2)	0.019 (2)	−0.0131 (19)
O3	0.095 (2)	0.0784 (18)	0.0610 (16)	−0.0093 (15)	0.0104 (15)	0.0029 (13)
C18	0.091 (3)	0.115 (3)	0.062 (2)	−0.040 (3)	0.002 (2)	−0.003 (2)

### Geometric parameters (Å, °)

**Table:**

S1—C1	1.673 (3)	C8—H8	0.9300
N1—C2	1.267 (3)	C9—H9A	0.9600
N1—N2	1.377 (3)	C9—H9B	0.9600
N2—C1	1.346 (3)	C9—H9C	0.9600
N2—H2	0.8600	C10—C11	1.449 (4)
N3—C1	1.337 (3)	C10—H10	0.9300
N3—N4	1.377 (3)	C11—C12	1.378 (4)
N3—H3	0.8600	C11—C16	1.379 (4)
N4—C10	1.272 (3)	C12—C13	1.369 (4)
O1—C6	1.367 (3)	C12—H12	0.9300
O1—C9	1.417 (4)	C13—C14	1.385 (4)
O2—C14	1.362 (3)	C13—H13	0.9300
O2—C17	1.429 (4)	C14—C15	1.359 (4)
C2—C3	1.455 (4)	C15—C16	1.376 (4)
C2—H2A	0.9300	C15—H15	0.9300
C3—C4	1.383 (4)	C16—H16	0.9300
C3—C8	1.384 (4)	C17—H17A	0.9600
C4—C5	1.379 (4)	C17—H17B	0.9600
C4—H4A	0.9300	C17—H17C	0.9600
C5—C6	1.379 (4)	O3—C18	1.380 (4)
C5—H5	0.9300	O3—H3A	0.8200
C6—C7	1.375 (4)	C18—H18A	0.9600
C7—C8	1.374 (4)	C18—H18B	0.9600
C7—H7	0.9300	C18—H18C	0.9600
			
C2—N1—N2	117.7 (2)	O1—C9—H9C	109.5
C1—N2—N1	117.9 (2)	H9A—C9—H9C	109.5
C1—N2—H2	121.0	H9B—C9—H9C	109.5
N1—N2—H2	121.0	N4—C10—C11	125.0 (3)
C1—N3—N4	122.9 (2)	N4—C10—H10	117.5
C1—N3—H3	118.5	C11—C10—H10	117.5
N4—N3—H3	118.5	C12—C11—C16	117.2 (3)
C10—N4—N3	113.5 (3)	C12—C11—C10	124.0 (3)
C6—O1—C9	117.8 (2)	C16—C11—C10	118.7 (3)
C14—O2—C17	117.2 (2)	C13—C12—C11	121.2 (3)
N3—C1—N2	116.1 (3)	C13—C12—H12	119.4
N3—C1—S1	119.4 (2)	C11—C12—H12	119.4
N2—C1—S1	124.5 (2)	C12—C13—C14	120.1 (3)
N1—C2—C3	119.8 (3)	C12—C13—H13	120.0
N1—C2—H2A	120.1	C14—C13—H13	120.0
C3—C2—H2A	120.1	C15—C14—O2	124.4 (3)
C4—C3—C8	118.0 (3)	C15—C14—C13	119.8 (3)
C4—C3—C2	120.8 (3)	O2—C14—C13	115.7 (3)
C8—C3—C2	121.2 (3)	C14—C15—C16	119.2 (3)
C5—C4—C3	121.9 (3)	C14—C15—H15	120.4
C5—C4—H4A	119.0	C16—C15—H15	120.4
C3—C4—H4A	119.0	C15—C16—C11	122.4 (3)
C6—C5—C4	119.1 (3)	C15—C16—H16	118.8
C6—C5—H5	120.4	C11—C16—H16	118.8
C4—C5—H5	120.4	O2—C17—H17A	109.5
O1—C6—C7	116.3 (3)	O2—C17—H17B	109.5
O1—C6—C5	124.1 (3)	H17A—C17—H17B	109.5
C7—C6—C5	119.6 (3)	O2—C17—H17C	109.5
C8—C7—C6	120.9 (3)	H17A—C17—H17C	109.5
C8—C7—H7	119.6	H17B—C17—H17C	109.5
C6—C7—H7	119.6	C18—O3—H3A	109.5
C7—C8—C3	120.5 (3)	O3—C18—H18A	109.5
C7—C8—H8	119.8	O3—C18—H18B	109.5
C3—C8—H8	119.8	H18A—C18—H18B	109.5
O1—C9—H9A	109.5	O3—C18—H18C	109.5
O1—C9—H9B	109.5	H18A—C18—H18C	109.5
H9A—C9—H9B	109.5	H18B—C18—H18C	109.5

### Hydrogen-bond geometry (Å, °)

**Table:**

*D*—H···*A*	*D*—H	H···*A*	*D*···*A*	*D*—H···*A*
O3—H3A···N1	0.82	2.55	3.171 (3)	134.
O3—H3A···S1	0.82	2.58	3.346 (3)	156.
N2—H2···O3^i^	0.86	2.45	3.174 (3)	142.
N3—H3···S1^ii^	0.86	2.61	3.446 (3)	165.
C2—H2A···O3^i^	0.93	2.51	3.300 (4)	143.

Symmetry codes: (i) −*x*+1, −*y*+2, −*z*; (ii) −*x*+1, −*y*+1, −*z*.

## References

[bb1] Affan, M. A., Chee, D. N. A., Ahmad, F. B. & Tiekink, E. R. T. (2010). *Acta Cryst.* E**66**, o555. 10.1107/S1600536810004241 PMC2983601 21580324

[bb2] Bruker (2007). *SMART* and *SAINT* Bruker AXS Inc., Madison, Wisconsin, USA.

[bb3] Camp, C., Mougel, V., Horeglad, P., Pcaut, J. & Mazzanti, M. (2010). *J. Am. Chem. Soc.* **132**, 17374–17377. 10.1021/ja1089364 21090629

[bb4] Loncle, C., Brunel, J. M., Vidal, N., Dherbomez, M. & Letourneux, Y. (2004). *Eur. J. Med. Chem.* **39**, 1067–1071. 10.1016/j.ejmech.2004.07.005 15571868

[bb5] Opstal, T. & Verpoort, F. (2003). *Angew. Chem. Int. Ed.* **42**, 2876–2879. 10.1002/anie.200250840 12833346

[bb6] Sheldrick, G. M. (1996). *SADABS* University of Göttingen, Germany.

[bb7] Sheldrick, G. M. (2008). *Acta Cryst.* A**64**, 112–122. 10.1107/S0108767307043930 18156677

